# Effect of migration on fertility and family planning: The case of Kayseri in Türkiye

**DOI:** 10.1097/MD.0000000000040716

**Published:** 2024-12-13

**Authors:** Mehmet Doğan, Fatma Özdemir, Şeyma Dağlituncezdi Çam

**Affiliations:** a Public Health, Vocational Health College, Erciyes University, Kayseri, Turkey; b Gynecology and Obstetrics, Faculty of Medicine, Erciyes University, Kayseri, Turkey; c Gynecology and Obstetrics, Kayseri City Hospital, Kayseri, Turkey.

**Keywords:** family planning, fertility, pregnancy, Syrian refugee, Türkiye

## Abstract

Many factors such as religious beliefs, cultural structure, the perspective of the host country toward refugees, and the course of the migration process can affect the family planning and fertility behaviors of refugees. This study aimed to determine the knowledge, attitudes, and behaviors of Syrian refugee pregnant women in the Kayseri province of Türkiye regarding fertility characteristics and family planning. This descriptive and cross-sectional study was conducted with 290 pregnant Syrian refugee women. In the study group, 58.3% of the participants were 18 years of age or younger when first married. In the study group, 39.0% had 4 or more pregnancies and the average number of pregnancies was 3.12 ± 1.60. The rate of refugee pregnant women who stated they were undecided regarding the knowledge, attitude, and behavior items about family planning ranged from 33.8% to 72.4%. Of the participants, 22.1% received family planning education. The mean number of pregnancies of the participating women was high. In addition, the rate of undecided respondents was high for the knowledge, attitude, and behavior items related to family planning. In conclusion, fertility behaviors should be planned by expanding family planning services. In this way, negative outcomes, especially in maternal and child health, can be prevented.

## 1. Introduction

Migration is as old as human history. Natural disasters, religion, poverty, wars, and conflicts cause people to move from 1 place to another.^[1]^ The United Nations High Commissioner for Refugees defines migration as people’s movement from their usual place of residence across an international border (international migration) or within a country.^[2]^ According to the 2022 United Nations World Migration Report, there are an estimated 281 million international migrants in the world. This figure corresponds to 3.6% of the global population.^[3]^

The current migration situation in Türkiye occurred when the civil war started in Syria in March 2011. However, efforts to introduce a law and an institution on migration management in Türkiye started long before the mass migration from Syria caused by the civil war. Developments in migration management in Türkiye date back to the “Accession Partnership Documents” of the European Union in 2001. The first legal regulation on migration in Türkiye was the enactment of the “Law on Foreigners and International Protection” in April 2013. In the law, migrants from Syria are governed under the “Temporary Protection” category. The relevant law defines those qualifying for temporary protection as “those who arrive at our borders en masse or individually with mass influxes, but whose international protection request cannot be evaluated individually.”^[4]^

According to the data of the Republic of Türkiye Ministry of Interior Directorate of Migration Management dated August 18, 2023, there are 3,307,882 registered Syrians under the temporary protection program. Of those under temporary protection in Türkiye, 760,797 (23.24%) are women between the ages of 15 and 49. In other words, approximately 1 out of every 4 Syrian refugees under temporary protection registered in Türkiye are women of childbearing age. Moreover, 41.4% (1,367,594) of the total refugees are in the 0 to 14 age group. The high number of women and children may mean that the number of births among Syrian refugees under temporary protection may be high. This interpretation is supported by the data. According to the data of the Directorate of Migration Management, the number of Syrian babies born in Türkiye between 2011 and 2021 exceeded 750,000.^[5]^

### 1.1. Fertility and family planning in pre-war Syria

According to the World Bank’s World Development Indicators 2012 report, the total fertility rate in Syria declined from 5.3 in 1990 to 2.9 in 2010. Despite this decline, Syria has the 6th-highest total fertility rate in the Arab world.^[6]^ In the Syrian Family Health Survey of 2009, 54% of married women were currently using contraception. Of these women, 22% used an intrauterine device (IUD), 8.9% the pill, 8.9% periodic abstinence, and 3.5% injectable drugs as birth control methods.^[7]^

### 1.2. Fertility and family planning among Turkish and Syrian women in Türkiye

According to the Turkish Demographic and Health Survey (TDHS) data, the total fertility rate in the Turkish population, which was 4.33 in 1978, gradually decreased to 2.3 in 2018, the most recent TDHS data.^[8]^ The Ministry of Health of the Republic of Türkiye reports that the total fertility rate decreased to 1.7 in 2021. According to 2018 TDHS data, 90.0% of married Turkish women used a family planning method at some time. Among these women, 77.0% used modern (effective) methods.^[9]^ The most commonly used methods were: coitus interruptus (58%), male condoms (49%), IUD (35%), and birth control pills (30%). The 2018 TDHS Syrian Migrant Sample data reported that the total fertility rate was 5.3. In the same report, 71.0% of Syrian married women used a family planning method at some time. Among these women, 54.0% used modern (effective) methods. The most common methods used by Syrian married women were: coitus interruptus (46%), IUD (34%), birth control pills (33%), and male condoms (13%).^[10]^

### 1.3. Why did family planning and fertility increase among Syrian refugees in Türkiye?

After Türkiye, Lebanon has the most Syrian refugees at a rate of 12.7% (840,000).^[11]^ A study conducted on the fertility preferences and behaviors of Syrian women in Lebanon found that Syrian refugee women desired to have fewer children.^[12]^ Despite the decrease in fertility in Lebanon, the increase in fertility in Türkiye is attributed to the provision of significant facilities for Syrian refugees to access health services and family medicine services similar to the Turkish people. Syrian refugees under temporary protection are not charged any fee for preventive, treatment, or emergency health services, which may be why family planning and fertility increased.^[13]^

### 1.4. Will Syrian refugees in Türkiye return to their country?

After approximately 12 years, the return of refugees to Syria is still an enigma. However, according to the results of the Syrians Barometer 2021 study, the rate of those who said “I do not think about returning to Syria in any way” increased from 16.7% in 2017 to 51.8% in 2019 and 77.8% in 2020.^[4]^ Considering the results of the Syrians Barometer 2021 study (conducted in 15 provinces), which is large-scale and representative of the population (in Türkiye), it can be stated that most Syrians will stay in Türkiye.

### 1.5. Do Syrian refugees affect the demographic structure in Türkiye?

Although Syrian refugees have spread across all cities in Türkiye, they are more concentrated in some cities. Istanbul, where approximately 540,000 Syrians are registered, has the largest number of Syrian refugees. In some provinces, the number of Syrian refugees is higher than the Turkish population. In Kilis province, the proportion of Syrian refugees in the total population is 73.5%. Kilis, with 107,000 Syrian refugees, has a total population of 145,000. In addition, 25.92% of the Hatay population (433,000 Syrians), 21.73% of the Gaziantep population (462,000 Syrians), and 20.07% of the Şanliurfa population (430,000 Syrians) are Syrian refugees.^[5]^

### 1.6. Aim of the study

Family planning has health benefits, especially for mothers and children, and social benefits in terms of improving nutrition, housing, education, and environmental conditions. In addition, family planning, which is related to population planning, affects the future population projections of countries. This study aims to determine the fertility characteristics of Syrian pregnant women and their knowledge, attitudes, and behaviors related to family planning. Accordingly, the study sought answers to the following questions.

What are the fertility characteristics of Syrian women in the study group?
What is the knowledge, attitudes, and behaviors of Syrian women in the study group about family planning?

## 2. Materials and methods

### 2.1. Study design

This descriptive and cross-sectional study was conducted with Syrian refugee pregnant women who visited the Obstetrics and Gynecology outpatient clinic of Kayseri Erciyes University Faculty of Medicine Hospitals between September and December 2022.

### 2.2. Sample size and sampling method

In the study, the sample size was calculated as 278 people for a 95% confidence interval, alpha = 0.05, power Beta = 0.80, and *d* = 0.10 effect size. However, 300 people were included in the study due to potential problems such as failure to fill out the questionnaire form completely.^[14]^ Overall, 10 pregnant women were excluded from the study due to incomplete questionnaires, and the data of 290 Syrian refugee pregnant women were evaluated.

### 2.3. Data collection

Study data were collected using a questionnaire form prepared by the researchers after reviewing the literature and included 39 questions, 19 about sociodemographic and fertility-related characteristics and 20 to determine knowledge, attitudes, and behaviors toward family planning. The questionnaire form was administered to pregnant women who visited the gynecology and obstetrics outpatient clinic and who volunteered to participate in the study. The questionnaire form was initially prepared in both Turkish and Arabic because the study was conducted with Syrian refugee pregnant women. The questionnaires were filled out through face-to-face interviews under the supervision of the researchers and with an Arabic-Turkish translator.

### 2.4. Data analysis

The study data obtained were transferred to the computer environment and evaluated using the SPSS 15.0 program (Chicago). Percentage and frequency distributions and standard deviation were calculated in statistical analysis.

### 2.5. Ethical approval statement

Participants in this study first gave verbal consent that they participated to volunteered. In addition, “Informed Volunteer Consent Form” consent was obtained from those who agreed to participate in the study, indicating that they allowed the publication of the study data. Ethical approval was obtained from Erciyes University Faculty of Medicine Non-interventional Clinical Research Ethics Committee (Decision No: 2022/487) to conduct the study.

## 3. Results

In the study group, 82.0% were between 19 and 24 years of age and the mean age was 25.12 ± 5.50. Of the participants, 7.6% were illiterate and 3.8% were university graduates. The age at first marriage was 18 years or below in 58.3% of the study group and the mean age at first marriage was 18.77 ± 3.42. Of the study group, 4.8% had at least 1 chronic disease; the most common chronic disease was goiter with a rate of 35.7%. The sociodemographic characteristics of the study group are shown in Table 1.

**Table 1:** Sociodemographic characteristics of the research group.

Sociodemographic characteristic	Number	%
Age		
18 and below	28	9.7
19–24	238	82.0
25 and over	24	8.3
Mean age (Min–Max)	25.12 ± 5.50 (Min**–**Max: 16–40)	
Educational status		
Illiterate	22	7.6
Literate without a diploma	66	22.8
Primary/middle school	124	42.7
Secondary school	67	23.1
Associate/graduate	11	3.8
Occupation		
Housewife	283	97.6
Self-employed	4	1.4
Teacher	2	0.7
Technician	1	0.3
Age at first marriage		
18 and below	169	58.3
19–24	103	35.5
25 and over	18	6.2
Mean age at first marriage (Min–Max)	18.77 ± 3.42 (Min**–**Max: 13–34)	
Chronic disease		
Yes	14	4.8
Present chronic disease (n = 14)		
Goiter	5	35.7
Rheumatism	3	21.4
Hypertension	3	21.4
Anemia	2	14.3
Coagulation disorder	1	7.2

Max = maximum, Min = minimum.

Of the study group, 18.3% were experiencing their first pregnancy. The mean number of pregnancies was 3.12 ± 1.60. The number of miscarriages, stillbirths, and abortions in the study group was 38.6%, 14.8%, and 8.6%, respectively. For the question “How old should a mother be before she becomes pregnant?” 95.2% of the participants and for the question “What age should a mother stop having children?” 100.0% stated that they did not know. The people who received the most family planning education in the study group were physicians with 31.2%. The 3 most commonly known family planning methods were IUD (31.4%), male condom (22.4%), and coitus interruptus (21.4%). The 3 most commonly used family planning methods in the study group were oral contraceptives (28.1%), coitus interruptus (28.1%), and IUD (21.1%). The characteristics of the research group related to childbirth and family planning are shown in Table 2.

**Table 2:** Characteristics of the research group related to childbirth and family planning.

Childbirth and family planning features	Number	%
Pregnancy number		
1	53	18.3
2	58	20.0
3	66	22.7
4 or more	113	39.0
Mean number of pregnancies (Min–Max)	3.12 ± 1.60 (Min–Max: 1–9)	
Have had a miscarriage?		
Yes	112	38.6
Number of miscarriages (n = 112)		
1	93	83.0
2	8	7.2
3	2	1.8
4 and more	9	8.0
Have had a stillbirth?		
Yes	43	14.8
Number of stillbirths (n* *= 43)		
1	40	90.0
2	3	7.0
Have had an abortion?		
Yes	25	8.6
Number of abortions (n = 25)		
1	24	96.0
2	1	4.0
How old should a mother be before she becomes pregnant?		
I don’t know	276	95.2
18 and over	14	4.8
What age should a mother stop having children?		
I don’t know	290	100.0
Person/place of family planning education (n* *= 64)		
Physician	20	31.2
Midwife/nurse	13	20.3
Radio/television	13	20.3
Newspaper/magazine/book	11	17.2
Relative/neighbor	7	11.0
Known family planning methods*		
IUD	90	31.0
Male condom	65	22.4
Coitus interruptus	62	21.4
Oral contraceptive	42	14.5
Lactational amenorrhea method (breastfeeding)	41	14.1
Cervical mucus or ovulation method	35	12.1
Vaginal Douche	35	12.1
Injection	27	9.3
Calendar method	24	8.3
Cervical palpation method	20	6.9
Diaphragm	20	6.9
Norplant/implant	19	6.6
Tubal ligation/vasectomy	16	5.5
Basal body temperature method	12	4.1
Symptothermal method	10	3.4
Spermicide	7	2.4
Family planning method used before the last pregnancy (n = 114)		
Oral contraception	32	28.1
Coitus interruptus	32	28.1
IUD	24	21.1
Male condom	19	16.6
Calendar method	7	6.1

IUD = intrauterine device, Max = maximum, Min = minimum.

* More than 1 option has been marked.

According to the responses to the questions on knowledge, attitudes, and behaviors related to family planning, the question with the highest rate of ambivalence was “Men who have their tubes tied lose their sexual power” (72.4%). The question with the highest rate of agreement was “God provides every child born” (56.2%). The question with the highest rate of disagreement was “The maxim ‘the woman’s primary duty is to bear children’ is true” (56.2%). Of the participants, 65.9% stated that they planned their last pregnancy and 49.7% stated that they wanted to become pregnant again after this pregnancy. Of the participating women, 77.9% stated that they had not received any family planning education. The distribution of the responses of the research group to the family planning knowledge, attitude, and behavior questions is shown in Table 3.

**Table 3:** Distribution of the responses of the research group to the questions on family planning knowledge, attitudes, and behavior.

Questions on family planning knowledge, attitude, and behavior	Yes, I agree		I’m undecided		No, I disagree	
	Number	%	Number	%	Number	%
The fact that there are many children in the family means that siblings will support each other in the future	149	51.4	98	33.8	43	14.8
The maxim “a woman’s primary duty is to bear children” is true	28	9.7	99	34.1	163	56.2
God provides every child born	163	56.2	102	35.2	25	8.6
I do not believe that getting pregnant at less than 2-year intervals is harmful to the child’s health	126	43.5	126	43.5	38	13.0
It is okay for a family to have many children because children can take care of each other	89	30.7	126	43.5	75	25.8
I do not believe that getting pregnant at less than 2-year intervals is harmful to maternal health	124	42.7	130	44.8	36	12.5
Pregnancy makes a woman attractive	80	27.6	145	50.0	65	22.4
I think there is no need to learn about contraception	73	25.1	154	53.2	63	21.7
It is feared that birth control pills can cause cancer	73	25.2	171	58.9	46	15.9
Birth control negatively affects sexual intercourse	48	16.5	163	56.2	79	27.3
Couples who use contraception have less sexual pleasure/desire	26	8.9	164	56.6	100	34.5
It is difficult for women who use contraception to have children later	39	13.4	171	59.0	80	27.6
The thread of the (IUD) spiral reduces sexual intercourse	40	13.8	193	66.5	57	19.7
Women who have their tubes tied lose their sexual power/are masculinized	30	10.4	205	70.7	55	19.0
Men who have their tubes tied lose their sexual power.	27	9.3	210	72.4	53	18.3
Wanting a child again after this pregnancy	144	49.7	10	3.4	136	46.9
Received information on family planning education*	64	22.1	–	–	226	77.9
Used family planning methods before the last pregnancy*	114	39.3	–	–	176	60.7
Became pregnant while using family planning methods (n = 114)*	31	27.2	–	–	83	72.8
Planned for the last pregnancy*	191	65.9	–	–	99	34.1

IUD = intrauterine device.

* Answered only with the answers “yes” or “no”.

## 4. Discussion

Among the strengths of this study is that it is one of the first studies on family planning in pregnant women in Turkey, the country with the largest number of Syrians, which is the largest refugee group in the world.

In a study based on focus group discussions conducted with Syrian women refugees in Lebanon, the participants stated that in Syria “girls are usually married before the age of 20, but the most common age of marriage is 14 to 15.” It was also stated that after the age of 20, girls are seen as spinsters.^[12]^ Similarly, in a study conducted in Türkiye based on focus group discussions conducted with Syrian women, the participants stated that in Syria, “girls get married starting from the age of 14, girls receive identity cards after the age of 15, parents make the marriage decision, and boys get married after military service (at the age of 19–20).”^[15]^ According to the results of the TDHS Syrian Migrant Sample study, the median age of marriage for Syrian women aged 20 to 49 years is 18.9.^[10]^ In the present study, the mean age at first marriage for the Syrian refugee pregnant women was 18.77 years (Table 1). Despite the low age at first marriage among Syrian refugee women in Türkiye, there was a relative increase compared to the age at first marriage in pre-war Syria. This may be due to the continued influence of the traditional and cultural structure of marriage and the low age at first marriage. The relative increase in the age of first marriage can be attributed to adaptation to a new society and the uncertainty caused by migration.

The age at first marriage means the beginning of the risk of pregnancy.^[16]^ According to the TDHS Syrian Migrant Sample study, 30.6% of Syrian women aged 15 to 19 had given live birth and 8.7% were pregnant with their first child. The adolescent fertility rate for women aged 15 to 19 years was 209 per 1000.^[10]^ In the present study, 58.3% of pregnant Syrian refugee women were 18 years of age or younger at their first marriage. Moreover, 7.6% of Syrian refugee pregnant women were illiterate, 22.8% were literate without a diploma, and only 3.8% were university graduates. Almost all Syrian pregnant women (97.6%) were housewives (Table 1). As a result of the low age for first marriage and having children at an early age, Syrian pregnant refugees may have had limited access to education and employment opportunities.

Although spontaneous abortions are purely medical conditions, induced abortions are important in terms of maternal health because they can negatively affect women’s health, reduce the possibility of having children in the future, and increase maternal mortality and perinatal mortality.^[10]^ Legally in Türkiye, pregnancies up to the 10th week of gestation can be terminated on request, even if there is no medical inconvenience for maternal health. After the 10th week, the pregnancy is terminated if the mother’s health is in danger or if the child will be disabled.^[16]^ In Syria, voluntary abortions are prohibited according to Articles 525-532 of the Penal Code, including for pregnancies resulting from rape.^[10]^ According to TDHS Syrian Migrant Sample data, a total of 32.0% of married Syrian women had at least 1 pregnancy loss, 27% of which were spontaneous and 5.0% were induced. In the present study, 38.6% of pregnant Syrian women had at least 1 miscarriage (Table 2). Compared to TDHS Syrian Migrant Sample data, the rate of miscarriage was higher in the present study. The study results were higher than the low rate in the TDHS Syrian Migrant Sample data. The reason for the high rate of miscarriage may be attributed to the fact that the study was conducted with pregnant women. The high rate of miscarriage in the study may be due to a lack of information on family planning services and the failure of birth control methods. In this study, the fact that 22.1% of Syrian pregnant women received information on family planning education and 27.2% of them became pregnant despite using family planning methods may support this result (Table 3).

Fertility behavior is known to impact maternal and childhood mortality. Young (under 18 years) or advanced (over 35 years) maternal age, high fertility (4 children and more), and short birth intervals (<24 months) are common risk factors.^[17]^ According to TDHS Syrian Migrant Sample data, the infant mortality rate among Syrian refugees is 22 per 1000 live births and the under 5 years mortality rate is 27 per 1000 live births. To summarize, 1 out of every 37 Syrian children in Türkiye dies before they reach their fifth birthday. Most of the deaths (81%) occur in the first year of life, and 44% of the deaths in the first year of life occur in the first month of life.^[10]^ According to data from the Ministry of Health, around the world, the mean maternal mortality is 223 per 100,000 live births and the maternal mortality rate in Türkiye in 2021 was 13.1.^[18]^ According to TDHS Syrian Migrant Sample data, if the child mortality risk rate of those not in any high-risk category is accepted as 1.00, the child mortality risk rate is 1.97 for those with a birth interval of <24 months.^[10]^ In this study, the rate of undecided respondents was high in many of the questions regarding knowledge, attitudes, and behaviors toward family planning. However, the rate of respondents who agreed with the questions “I do not believe that getting pregnant at less than 2 years’ intervals is harmful for maternal health” and “child health” was high (42.7% and 43.5%, respectively). The high rate of agreement with these questions may be due to a lack of knowledge about family planning. In fact, family planning aims to enable spouses to have as many children as they want by determining the birth intervals in a way that does not affect the health of the woman.

Family planning ensures that spouses have the number of children they want, at the time they want, at intervals appropriate for maternal and child health.^[19]^ Families are free to have children and can have as many children as they want, can look after, and raise with their own will. The important thing is that families make decisions consciously and responsibly.^[16]^ Syria is a country where most of the population is Muslim. Islam considers that family planning is acceptable provided that the spouses agree and time periods are set between the children to be born. As in the definition of family planning, one of the conditions in Islam is that families must provide a healthy upbringing for their children.^[20]^ In this study, 56.2% of the Syrian refugee pregnant women stated that they agreed with the idea “God provides every child born” (Table 3). As can be seen, both the definition of family planning and the view of Islam on family planning are clear. Education programs on family planning should be given to all Syrian refugees, especially Syrian women. Providing these programs supported with the rules of Islam will increase the quality and acceptability of the service provided.

In a study conducted based on focus interviews with Syrian refugee women in Türkiye, the participants stated that husbands have more say in the number of children and that they give birth to children for their husbands. These focus interviews revealed how males are influential in women’s decisions on pregnancy.^[15]^ While the man wants to have children for the continuation of his lineage and family name, the woman tries to gain the respect of the man and his family by giving birth to many children. However, the principles of ensuring gender equality, empowering women, and ensuring that women have a say in their fertility were accepted at the International Conference on Population and Development held in Cairo in 1994.^[21]^ In this study, 56.2% of Syrian refugee pregnant women stated that they disagreed with the item “The maxim that the primary duty of women is to bear children” (Table 3). This may mean that there has been a change in Syrian refugee women’s participation in decisions about their pregnancy. It is also important in terms of recognizing that family planning is not a women’s issue and that men are also included in family planning. The fact that the second most common family planning method used before the last pregnancy was the male condom was an important finding that explains this situation more clearly (Table 2).

## 5. Conclusion

Syrian refugee pregnant women in the study group had a low age at first marriage and a high average number of pregnancies. The number of Syrian refugees who received family planning education was low. The rate of undecided respondents was high in knowledge, attitude, and behavior questions about family planning. The reason for being undecided about family planning is due to a lack of information. Education programs should be conducted for all Syrian refugees, especially for Syrian women refugees, to overcome the lack of knowledge about family planning.

## Author contributions

**Conceptualization:** Mehmet Doğan, Fatma Özdemir, Şeyma Dağlituncezdi Çam.

**Data curation:** Mehmet Doğan, Fatma Özdemir, Şeyma Dağlituncezdi Çam.

**Formal analysis:** Mehmet Doğan.

**Methodology:** Mehmet Doğan, Fatma Özdemir, Şeyma Dağlituncezdi Çam.

**Project administration:** Mehmet Doğan.

**Writing – original draft:** Mehmet Doğan, Fatma Özdemir.
